# Poison-Exon Inclusion in DHX9 Reduces Its Expression and Sensitizes Ewing Sarcoma Cells to Chemotherapeutic Treatment

**DOI:** 10.3390/cells9020328

**Published:** 2020-01-31

**Authors:** Ramona Palombo, Veronica Verdile, Maria Paola Paronetto

**Affiliations:** 1 Laboratory of Cellular and Molecular Neurobiology, IRCCS Fondazione Santa Lucia, 00143 Rome, Italy; r.palombo@hsantalucia.it (R.P.); v.verdile@studenti.uniroma4.it (V.V.); 2 Department of Movement, Human and Health Sciences, Università degli Studi di Roma “Foro Italico”, Piazza Lauro de Bosis, 15, 00135 Rome, Italy

**Keywords:** DHX9, alternative splicing, Ewing sarcoma, chemoresistance

## Abstract

Alternative splicing is a combinatorial mechanism by which exons are joined to produce multiple mRNA variants, thus expanding the coding potential and plasticity of eukaryotic genomes. Defects in alternative splicing regulation are associated with several human diseases, including cancer. Ewing sarcoma is an aggressive tumor of bone and soft tissue, mainly affecting adolescents and young adults. DHX9 is a key player in Ewing sarcoma malignancy, and its expression correlates with worse prognosis in patients. In this study, by screening a library of siRNAs, we have identified splicing factors that regulate the alternative inclusion of a poison exon in *DHX9* mRNA, leading to its downregulation. In particular, we found that hnRNPM and SRSF3 bind in vivo to this poison exon and suppress its inclusion. Notably, DHX9 expression correlates with that of SRSF3 and hnRNPM in Ewing sarcoma patients. Furthermore, downregulation of SRSF3 or hnRNPM inhibited DHX9 expression and Ewing sarcoma cell proliferation, while sensitizing cells to chemotherapeutic treatment. Hence, our study suggests that inhibition of hnRNPM and SRSF3 expression or activity could be exploited as a therapeutic tool to enhance the efficacy of chemotherapy in Ewing sarcoma.

## 1. Introduction

Alternative splicing is a sophisticated mechanism by which exons are joined in different combinations to generate multiple mRNA variants, thus fine-tuning gene expression programs [1,2]. Regulation of alternative splicing enacts cell growth, differentiation, and survival [3] and is deeply involved in the development and progression of human pathological conditions [4].

A surveillance mechanism of regulation that limits the expression of aberrant transcripts in the cells is nonsense-mediated mRNA decay (NMD) [5], which is triggered by the presence of a premature stop codon (PTC) in non-last exons. Such “poison” cassette exons are normally skipped, while their inclusion in the mature mRNA targets it to NMD [6,7,8]. Indeed, the presence of a PTC located more than 50 nucleotides upstream of the final exon–exon junction is thought to signal the premature or aberrant nature of a transcript [6,7,8]. In addition to poison cassette exons, other classes of alternative splicing events, such as alternative 5′- and 3′-splice sites or intron retention, also contribute to the alternative splicing-NMD post-transcriptional regulation [9], thus limiting the accumulation of unproductive splicing variants. Noteworthy, the expression of many RNA binding proteins (RBPs) involved in splicing regulation, such as the serine/arginine-rich (SR) proteins, is fine-tuned by NMD [6,8].

Changes in the RNA polymerase II (RNAPII) elongation rate contribute to the regulation of NMD-linked alternative splicing, thus coordinating the cellular requirements of splicing factors and RBPs in response to internal and external cues. RNAPII slowing down and occupancy over the intronic regions flanking the regulated exons favor the recognition of suboptimal splice sites, such as those in poison exons [10]. We previously identified a poison exon (exon 6A) in the *DHX9* gene, whose inclusion targets the transcript to NMD [11]. Inclusion of exon 6A is normally repressed, thus insuring high expression levels of DHX9. However, reduction in the RNAPII elongation rate within the DHX9 transcription unit favors exon 6A inclusion and targets the transcript to NMD [11]. Both UV light irradiation and etoposide treatment induced this event by slowing down the RNAPII [11], with the consequent decrease in DHX9 expression, thus leading to higher sensitivity of Ewing sarcoma cells to genotoxic stress [11,12]. Nevertheless, the mechanism by which exon 6A inclusion is normally repressed in Ewing sarcoma cells is currently unknown.

DHX9 is a member of the DExH subgroup of RNA helicases, which play important roles in several aspects of RNA metabolism [12]. DHX9 is involved in the regulation of gene expression by acting as a scaffold for the interaction of breast cancer 1 (BRCA1) [13] and cyclic adenosine monophosphate (AMP) response element-binding protein-binding protein (CBP) [14] with the RNAPII holoenzyme, thus modulating their activity and regulating transcription. Moreover, DHX9 is involved in the maintenance of genomic stability [15,16,17]. In Ewing sarcoma, DHX9 forms a complex with the EWS-FLI1 oncoprotein and modulates EWS-FLI1-dependent transcription [18]. In particular, the functional interaction between DHX9 and EWS-FLI1 enhances the engagement of the transcriptional machinery at responsive promoters, induces local changes in chromatin structure, and unwinds the DNA. DHX9 also interacts with the RBP Sam68 and with the promoter-associated noncoding RNA *pncCCND1b* to form an RNA-protein complex inhibiting *CCND1* transcription in Ewing sarcoma cells [19].

The EWS-FLI1/DHX9 complex represents a good therapeutic target for Ewing sarcoma [11,18,20,21,22,23]. Thus, understanding the regulation of the *DHX9* poison-exon 6A inclusion might pave the way for novel splicing-directed strategies to inhibit *DHX9* gene expression and EWS-FLI1 oncogenic activity. Herein, we screened a library of siRNAs targeting RBPs to identify factors that regulate *DHX9* alternative splicing. We identified hnRNPM and SRSF3 as key factors required to suppress exon 6A inclusion and maintain high DHX9 expression in Ewing sarcoma cells. Importantly, downregulation of SRSF3 or hnRNPM sensitized Ewing sarcoma cells to doxorubicin, a genotoxic agent used in Ewing sarcoma chemotherapy. Therefore, our study suggests that inhibition of hnRNPM or SRSF3 expression could be exploited as a therapeutic tool in Ewing sarcoma.

## 2. Materials and Methods

### 2.1. Cell Cultures and Drug Treatment

Ewing sarcoma cell lines TC-71 (RRID: CVCL_2213 and SK-N-MC RRID: CVCL_0530) were purchased from DSMZ (Braunschweig, Germany). LAP-35 (RRID: CVCL_A096) was a generous gift from Drs. Katia Scotlandi and Cristina Manara. The absence of mycoplasma contamination was verified every two months by PCR analysis. Cells were maintained in culture in Iscove’s modified Dulbecco’s medium (IMDM) (GIBCO—Thermo Fisher Scientific, Waltham, USA, Massachusetts), supplemented with 10% fetal bovine serum, and penicillin and streptomycin (GIBCO) and maintained at 37 °C in humidified 5% CO_2_ atmosphere. For doxorubicin treatment, Ewing sarcoma cells were treated for the indicated time with either DMSO or the indicated concentrations of doxorubicin (ranging from 0.1 nM to 150 nM).

### 2.2. Transfections

Lipofectamine RNAiMax reagent (Thermo Fisher Scientific, Waltham, MA, USA) was used for siRNA transfections. Briefly, 20,000 TC-71 cells were subjected to double pulse of reverse-transfection by using 2 μL of Lipofectamine RNAiMAX, and cells were collected or re-plated for further experiments 24 h after the last pulse of transfection. siRNAs and primers oligonucleotides were purchased from Sigma–Aldrich (Milan, Italy). Sequences are listed in Supplementary Tables S1 and S2, respectively.

### 2.3. SDS–PAGE and Western Blot Analyses

For protein extract preparation, cells were washed twice with ice-cold phosphate-buffered saline (PBS), resuspended in RIPA lysis buffer (150 mM NaCl, 50 mM Tris-HCl pH 7.5, 2 mM EDTA, 0.1 % in sodium dodecyl sulfate (SDS), 0.5% sodium deoxycolate,1mM dithiothreitol, 0.5 mM Na-orthovanadate, 1%, 10 mM β-glycerolphosphate, 10 mM sodium fluoride, 1% NP-40 and Protease-Inhibitor Cocktail (Sigma–Aldrich)) and kept on ice for 10 min. Soluble protein extracts were separated by centrifugation at 12,000 rpm for 10 min and diluted in Laemlli sample buffer. The obtained cell lysates were resolved on SDS–polyacrylamide gels (SDS-PAGE) and transferred on PVDF membrane Hybond TM-P (Amersham Bioscience, Buckinghamshire, UK). Membranes were saturated with 5% BSA at room temperature and incubated with the following primary antibodies at 4 °C overnight: mouse GAPDH (SC-32233), rabbit DHX9 (SC-66997), mouse SRSF1 (SC-33652), mouse HNRNPM (SC-20002), rabbit HNRNPK (SC-25373), mouse FUS (SC-47711), and β-actin (SC-47778) from Santa Cruz Biotechnology Inc. (Dallas, TX, USA), and mouse SRSF3 (Abnova, Taipei, Taiwan, (H00006428-MO8)). Secondary anti-mouse or anti-rabbit IgGs conjugated to horseradish peroxidase (Amersham Bioscience) were incubated with the membranes for 1 h at room temperature at a 1:10,000 dilution. Immunostained bands were detected by a chemiluminescent method (Thermo Fisher Scientific).

### 2.4. Real-time Quantitative PCR Analyses (RT-qPCR)

RNA was isolated and DNase digested using either Trizol (Thermo Fisher Scientific) or RNeasy kit (Qiagen, Hilden, Germany). Total RNA (1 µg) was reverse transcribed by using M-MLV Reverse Transcriptase (Promega, Madison, WI, USA) following the manufacturer’s instructions. RT reaction was used as a template together with the different primers listed in Supplementary Table S2. Primers were designed using Primer 3 Plus (http://www.bioinformatics.nl/cgi-bin/primer3plus/primer3plus.cgi) and Primer-Blast using the reference and the alternative RefSeq accession numbers. For RT-qPCR, the primers were designed such that their annealing temperature was 60 °C, generating single-amplification products in the range of 60- to 120-base-pairs (bp) long. PCR amplification was carried out with 1 μL of the 1:10 diluted reverse transcription sample with 10 μL of 2X SYBR Green Master Mix (Roche, Basel, Germany) and 4 pmol of specific gene primer pairs in a 20 μL total volume in 96-well microtiter plates. PCR reactions were run in triplicates on a LightCycler 480 system (Roche). Each experiment was performed at least in triplicate; data are represented as the mean ± standard deviation (SD). For all experiments, no-RT controls have been performed.

### 2.5. CLIP Assays

Cross-linked and immunoprecipitation (CLIP) assays were performed as previously described [24]. In brief, TC-71 cells were irradiated once with 400 mJ/cm^2^ in a Stratalinker 2400 at 254 nm. Cell suspension was centrifuged at 4000 rpm for 3 min, and the pellet was incubated for 10 min on ice in lysis buffer (100 mM NaCl, 10 mM MgCl_2_, 30 mM Hepes pH 7.6, 2 mM EDTA pH 8, 10% Glycerol, 0.5% Tryton-X100, RNase inhibitor, cocktail protease inhibitor (Sigma–Aldrich), 1 mM dithiothreitol, 0.5 mM Na-ortovanadate, 1%, 10 mM β-glycerolphosphate, and RNase inhibitor (Promega). Samples were briefly sonicated and incubated with 10 μL of 1/1000 RNase I (Ambion, AM2295, Thermo Fisher Scientific) dilution and 2 μL Turbo DNase (Ambion, AM2238, Thermo Fisher Scientific) for 3 min at 37 °C shaking at 1100 rpm, and then centrifuged at 13,000 rpm for 10 min at 4 °C. One point five milligrams of the extract was immunoprecipitated overnight using anti-hnRNPM, anti-SRSF1, anti-SRSF3, anti-hnRNPK, anti-FUS antibodies or purified rabbit or mouse IgGs (negative control) in the presence of protein A/G magnetic Dynabeads (Life Technologies–Thermo Fisher Scientific). Immunoprecipitates were incubated overnight at 4 °C under constant rotation. After stringent washes with high salt buffer (300 mM NaCl, 10 mM MgCl2, 30 mM Hepes pH 7.6, 2 mM EDTA pH 8, 10% Glycerol, 0.5% Tryton-X100) beads were equilibrated with PK buffer (100 mM Tris-HCl, pH 7.4, 50 mM NaCl, 10 mM EDTA). An aliquot (10%) was kept as a control of immunoprecipitation, while the rest was treated with 50 µg Proteinase K and incubated for 20 min at 37 °C shaking at 1100 rpm. Seven molars of urea was added to the PK buffer, and incubation was performed for a further 20 min at 37 °C and 1100 rpm. The solution was collected, and phenol/CHCl3 (Ambion, 9722—Thermo Fisher Scientific) was added. After incubation for 5 min at 30 °C shaken at 1100 rpm, phases were separated by spinning for 5 min at 13,000 rpm at room temperature. The aqueous layer was transferred into a new tube and precipitated by the addition of 0.5 μL glycoblue (Ambion, 9510—Thermo Fisher Scientific), 3 M sodium acetate pH 5.5, and 100% ethanol. After mixing, the solution containing retained RNA was precipitated overnight at −20 °C. Purified RNA was used for qPCR analysis. Primers used are listed in Supplementary Table S2.

### 2.6. MTS Proliferation Assay

Cell proliferation was determined using the Cell Titer A96 3-(4,5-dimethylthiazol-2-yl)-5-(3-carboxymethoxyphenyl)-2-(4-sulfophenyl)-2*H*-tetrazolium, inner salt (MTS) method according to the manufacturer’s instructions (Promega) by plating 5,000 cells/well in 96-well culture plates.

### 2.7. Colony Formation Assay

Cells were plated in 35 mm plates at a density of 3,000 cells/plate. After one day, cells were transfected with either scrambled or siRNAs oligonucleotides. Twenty-four hours after transfection, cells were treated with the indicated reagents and incubated at 37 °C in a humidified atmosphere containing 5% CO_2_ for 12 days, replacing medium every two days. At the end of the incubation period, cells were washed with PBS, fixed in methanol for 10 min at RT, and stained 30 min at room temperature with 0.05% Crystal Violet in distilled water on a rotating shaker. After staining, cells were washed twice with tap water and air-dried overnight. Clones were counted, and the percentage of control was calculated.

### 2.8. Patient Datasets

**Figure 1:**
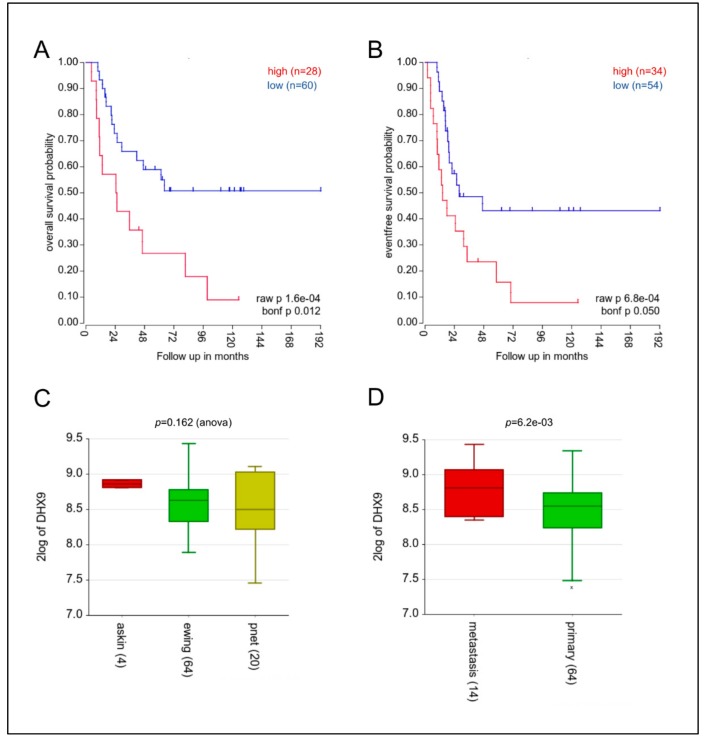
*DHX9* expression is a prognostic factor for Ewing sarcoma malignancy. Kaplan–Meier plots of overall (**A**) and event-free survival (**B**) probabilities generated by gene expression profiling ([25]; GSE17679) in R2 Genomics (https://hgserver1.amc.nl/cgi-bin/r2/main.cgi). The dataset analyzed is composed of 64 Ewing sarcoma, 4 Askin, and 20 PNET (Peripheral Primitive Neuro-Ectodermal Tumors) patients. *P*-values and Bonferroni post-hoc corrections are indicated on the bottom. (**C**) The box plot shows DHX9 expression in Ewing sarcoma (64 patients), Askin tumor (4 patients), and PNET (20 patients). Statistical analysis was performed by one-way ANOVA. (**D**) DHX9 expression was monitored by stratifying the previous dataset in primary and metastatic tumors ([25] GSE17679). Statistical analysis was performed by Student’s *t*-test (*p*-value is indicated on the top of the graph).

**Figure 2:**
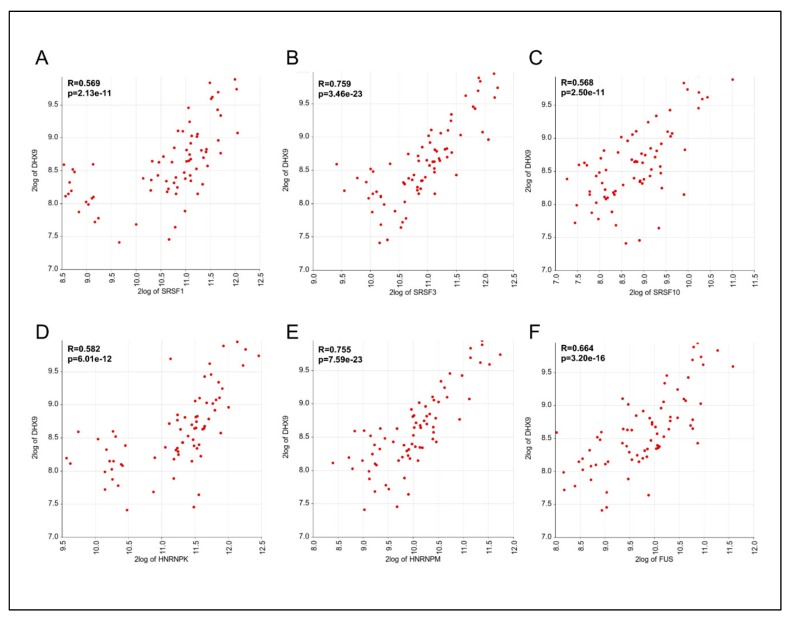
*SRSF3* and *hnRNPM* expression in ES patients exhibit a significant correlation with *DHX9* expression. Pearson correlation analysis on Ewing sarcoma patients, performed between the expression of DHX9 and SRSF1 (**A**), SRSF3 (**B**), SRSF10 (**C**), hnRNPK (**D**), hnRNPM (**E**), or FUS (**F**). Values are expressed as base 2-logarithm. In each panel, the correlation value (R) and the relative *p*-value are indicated.

The patient dataset used for the analyses in Figure 1 and Figure 2 derives from public available Affymetrix Human Genome U133 Plus 2.0 microarray data of a total of 88 Ewing sarcoma samples (gse17679; Ethical Review Board of Helsinki University Central Hospital no. 329 HUS/E0/05 and 328 HUS/W0/05) [25]. Data are available online at the following link [26]. Detailed patient information is available at the following link: https://www.ncbi.nlm.nih.gov/geo/geo2r/?acc=GSE17679.

### 2.9. Bioinformatic Analysis

Event-free and overall survival analyses were performed on a dataset composed of 64 Ewing sarcoma, 4 Askin, and 20 PNET (Peripheral Primitive Neuro-Ectodermal Tumors) patients (dataset: GSE17679 [25]) using R2: Genomics Analysis and Visualization Platform website (http://r2.amc.nl).

### 2.10. Statistical Analysis

Statistical analysis for biological assays was performed by using Graphpad Prism software to calculate EC_50_, 2-tailed unpaired *t*-test or one-way or two-way ANOVA, as appropriate. All data are presented as mean ± S.D.

## 3. Results

### 3.1. DHX9 Expression Correlates with Worse Prognosis in Ewing Sarcoma Patients

Given the functional relevance of DHX9 in genomic instability and in cancer [12], we asked whether its expression represents a prognostic factor in Ewing sarcoma. Analysis of event-free survival and overall survival in a dataset comprising 64 Ewing sarcoma, 4 Askin, and 20 perypheral primitive neuroectodermal tumors [25], which all belong to the family of Ewing tumors, showed that high DHX9 expression correlates with worse prognosis of the patients (Figure 1A,B). No significant differences in DHX9 expression were observed between Askin, Ewing, and PNET tumors (Figure 1C). However, we observed a significantly higher expression of DHX9 in metastatic tumors versus primary tumors (*p* = 6.2 × 10^3^). These results support a role of DHX9 in Ewing sarcoma malignancy and highlight its potential contribution to the metastatization process (Figure 1D).

### 3.2. A siRNA Library Identifies Regulators of DHX9 Alternative Splicing

**Figure 3:**
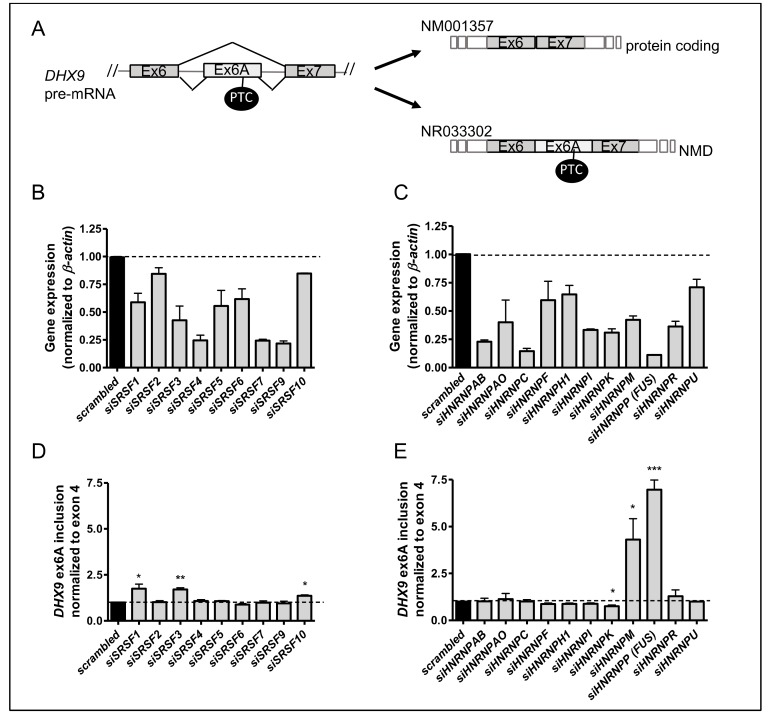
Regulation of *DHX9* alternative splicing. (**A**) Schematic representation of the alternative splicing of exon 6A in *DHX9* pre-mRNA. Exclusion of the poison exon 6A in *DHX9* mRNA leads to the main transcript (NM001357) encoding the full-length DHX9 protein (upper part). Exon 6A inclusion leads to the alternative noncoding transcript NR033302, containing a premature stop codon (PTC) and targeted to the NMD machinery. (**B**–**E**) Histograms represents RT-qPCR analysis of a siRNA library to downregulate the expression of SR proteins (**B**) (one-way ANOVA *p*-value < 0.0001; Bonferroni correction for *siSRSF6 p*-value < 0.05; *siSRSF1* and *siSRSF5 p*-value < 0.005; *siSRSF3*, *siSRSF4*, *siSRSF7*, *siSRSF9 p*-value < 0.001, *siSRSF2* and *siSRSF10 p*-value > 0.05) or hnRNPs (**C**) (ANOVA *p*-value < 0.0001; Bonferroni correction for *sihnRNPF* and *sihnRNPH1 p*-value < 0.05; *siHNRNPAB, siHNRNPAO, siHNRNPC*, *siHNRNPK*, *siHNRNPI*, *siHNRNPM, siHNRNPP,* and *siHNRNPR p*-value < 0.001, *siHNRNPU p*-value > 0.05). *DHX9* Exon 6A (Ex6a) inclusion was monitored by RT-qPCR and normalized to the constitutive exon 4 (**D**,**E**). Reported values represent the average (± S.D.) of at least three independent experiments. Statistical analysis was performed by one-way ANOVA, with *p*-value < 0.0001 (**D**,**E**) and with Bonferroni post-hoc test. (*p*-value: *** < 0.001, ** < 0.01, *< 0.05).

DHX9 pre-mRNA can undergo alternative splicing to produce either a transcript translated into the full-length protein (NM001357) or a noncoding transcript (NR033302), containing the poison exon 6A, which is targeted to NMD (Figure 3A; [11]). To identify endogenous regulators of this alternative exon, we screened a siRNA library for splicing factors belonging to the serine–arginine (SR) rich and the heterogeneous nuclear ribonucleoprotein (hnRNP) families, which represent the main regulators of alternative splicing [27,28]. Oligonucleotides to knockdown either SR proteins or hnRNPs were transfected in TC-71 Ewing sarcoma cells, and RNA was extracted 48 h later. RT-qPCR analysis was performed to verify the downregulation of the RBP transcripts (Figure 3B,C) and to assess the level of exon 6A inclusion in cells transfected with the siRNA library. The level of exon 6A inclusion was normalized for both a constitutive exon (DHX9 exon 4; Figure 3D,E) and the exon junction exon 6-exon 7 (Supplementary Figure S1A,B). Increased inclusion of exon 6A was observed upon knockdown of SRSF1, SRSF3, SRSF10, HNRNPM, and HNRNPP (FUS), with respect to cells transfected with a control siRNA. On the other hand, knockdown of the HNRNPK transcript promoted further skipping of the alternative exon. Downregulation of the most significant regulators (SRSF1, SRSF3, hnRNPM, hnRNPK, and FUS) was then confirmed at the protein level by Western blot analysis (Supplementary Figure S1C). Given the high similarity between FUS and EWS, we also ascertained that FUS downregulation did not affect neither EWS nor EWS-FLI1 expression (Supplementary Figure S1D). Collectively, these results suggest that several specific SR proteins and hnRNPs contribute to the regulation of DHX9 alternative splicing in TC-71 cells.

### 3.3. CLIP Assay Unveils Direct Binding of Specific SR Proteins and HnRNPs to DHX9 Pre-mRNA

**Figure 4:**
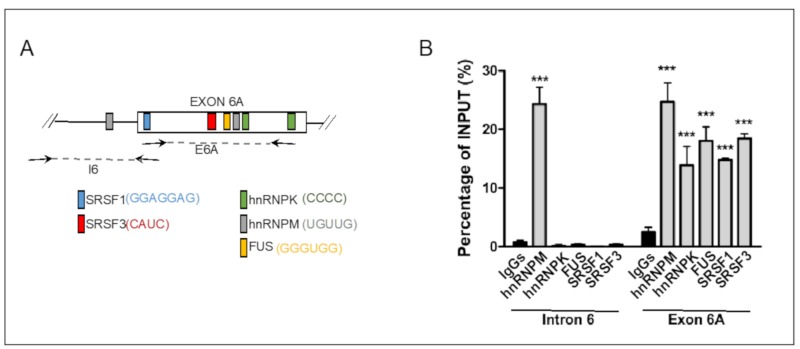
*SRSF1, SRSF3, hnRNPK, hnRNPM,* and *FUS* bind *DHX9* exon 6A. (**A**) In silico analysis was performed using the Splice Aid database to identify putative consensus motifs for RBPs in the exon 6A sequence. In (A), schematic representation of *DHX9* alternative exon 6A, with the position of SRSF1 (blue), SRSF3 (red), hnRNPK (green), hnRNPM (grey), and FUS (yellow) binding sites. Arrows indicate the primers’ positions used for the amplicons along the sequence. (**B**) TC-71 cells were UV-crosslinked, and protein-RNA extracts were immunoprecipitated with either control IgGs or hnRNPM, hnRNPK, FUS, SRSF1, and SRSF3 antibodies. Experiments were performed at least three times. Histograms show RT-qPCR analysis of the cross-linked and immunoprecipitation (CLIP) assay to analyze the binding of the indicated RNA binding proteins (RBPs) to intron 6 and exon 6A of *DHX9* pre-mRNA. Results are expressed as a percentage of input (± S.D.). Statistical analysis was performed by one-way ANOVA with Bonferroni post-hoc test (*p*-value: *** < 0.001).

SR proteins and hnRNPs generally regulate splice site selection by direct binding to the pre-mRNA [2,27]. In silico analysis was performed by querying the Splice Aid database (http://193.206.120.249/splicing_tissue.html) to identify potential binding sites for the main regulators of exon 6A splicing (SRSF1, SRSF3, SRSF10, hnRNPK, hnRNPM, and FUS) in the region of the pre-mRNA encompassing the regulated exon. Remarkably, we found that DHX9 exon 6A contains consensus motifs for all of them, except SRSF10 (Figure 4A). To verify the binding of these factors in live cells, we performed cross-linked and immunoprecipitation (CLIP) experiments of SRSF1, SRSF3, hnRNPM, hnRNPK, and FUS from UV-cross-linked TC-71 cell extracts. RT-qPCR analysis of the RNA associated with these splicing factors revealed that all of them were able to bind in vivo to the DHX9 pre-mRNA within the exon 6A region, although with different affinities (Figure 4B). On the other hand, as predicted from the in silico analysis (Figure 4A), only hnRNPM was also able to bind in the upstream intron 6 region (Figure 4B). These experiments indicate that SRSF1, SRSF3, hnRNPM, hnRNPK, and FUS are endogenous regulators of DHX9 exon 6A alternative splicing in TC-71 cells.

### 3.4. Expression of DHX9, HNRNPM, and SRSF3 is Positively Correlated in Ewing Sarcoma Patients

To test whether the expression of splicing factors involved in exon 6A regulation was correlated with DHX9 mRNA levels in Ewing sarcoma patients, we performed Pearson correlation analyses using a dataset of 64 Ewing sarcoma patients, 4 Askin, and 20 PNET tumors. A significant correlation was observed with all the splicing regulators of exon 6A, with highest R values observed with SRSF3 (R = 0.759, *p* = 3.46 × 10^−23^ and HNRNPM (R = 0.755, *p* = 7.59 × 10^−23^) transcripts. Remarkably, RBPs that did not regulate DHX9 splicing in our screening, such as hnRNPL and SRSF5 (Figure 4), did not correlate with DHX9 expression (Supplementary Figure S2). Since high DHX9 expression correlates with poor prognosis (Figure 1), these results suggest that regulation of DHX9 expression by SRSF3 and hnRNPM could represent a prognostic factor in Ewing sarcoma pathogenesis.

### 3.5. Depletion of SRSF3 and HnRNPM Affects the Expression of EWS-FLI1 Target Genes

**Figure 5:**
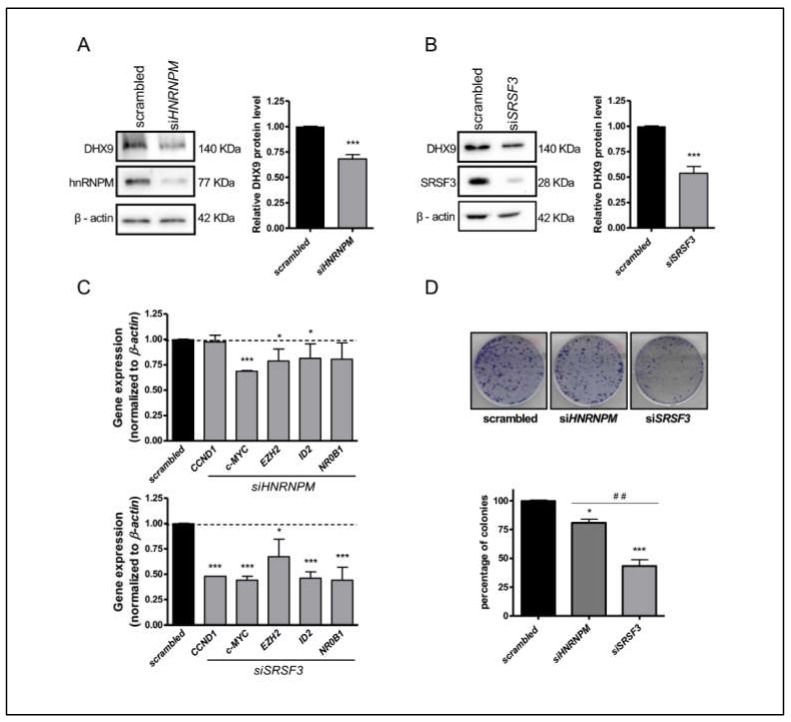
*DHX9* exon 6A inclusion affects Ewing sarcoma cells’ viability and proliferation. Western blot analysis of TC-71 cell extracts knocked down for either hnRNPM (**A**) or SRSF3 (**B**). DHX9 protein levels were monitored and normalized to β-actin. Histogram represents the densitometric analysis of DHX9 expression from three independent experiments (average ± S.D.). Statistical analysis was performed by Student *t*-test (*p*-value *** < 0.001). (**C**) RT-qPCR analysis to detect the expression of EWS-FLI1 target genes (*CCND1, c-MYC, EZH2, ID2, NR0B1*) upon silencing of *HNRNPM* (upper part) and *SRSF3* (lower part). (**D**) Representative clonogenic assay of cells transfected with either scrambled or siRNA oligonucleotides targeting *HNRNPM* and *SRSF3* transcripts. On the bottom, the bar graph shows the percentage of colonies in each condition versus scrambled. Student *t*-test was used for statistical analysis (*p*-value: *** < 0.001, *<0.05; ##<0.01 *siHNRNPM vs siSRSF3*).

Since the inclusion of exon 6A targets the *DHX9* transcript to NMD, we performed Western blot analysis to evaluate whether SRSF3 and hnRNPM knockdown affected DHX9 expression. A significant decrease (35% for siHNRNPM and 50% for siSRSF3) in the DHX9 protein level was observed upon the silencing of these two splicing factors in TC-71 cells (Figure 5A,B). Similar results were also obtained in SK-N-MC and LAP-35 Ewing sarcoma cells (Supplementary Figure S3), even though HNRNPM and SRSF3 knockdown in LAP-35 weakly affected DHX9 alternative splicing.

As previously reported [11,18,20,21,22,23], disruption of the DHX9-EWS-FLI1 interaction strongly impacts on the EWS-FLI1-driven transcriptional program in Ewing sarcoma cells. Thus, we asked whether knockdown of SRSF3 and hnRNPM had a similar effect on EWS-FLI1 target genes. RT-qPCR analysis showed that expression of c-MYC, EZH2, and ID2, was downregulated upon silencing of the two RBPs, with stronger effects elicited by depletion of SRSF3. Moreover, expression of NR0B1 and CCND1 was affected only by SRSF3 depletion (Figure 5C).

To evaluate the effect of these splicing factors on Ewing sarcoma cell proliferation, we performed colony assays with cells transfected with control siRNA or with si-*HNRNPM* and si-*SRSF3* oligonucleotides. Depletion of hnRNPM, and even more of SRSF3, caused a significant reduction in the colony formation potential of Ewing Sarcoma cells (Figure 5D). These findings demonstrate that hnRNPM and SRSF3 promote DHX9 expression and Ewing sarcoma cells proliferation and viability, suggesting that targeting their expression or activity may have beneficial effects.

### 3.6. Depletion of SRSF3 and hnRNPM Increases Doxorubicin Sensitivity of Ewing Sarcoma Cells

**Figure 6:**
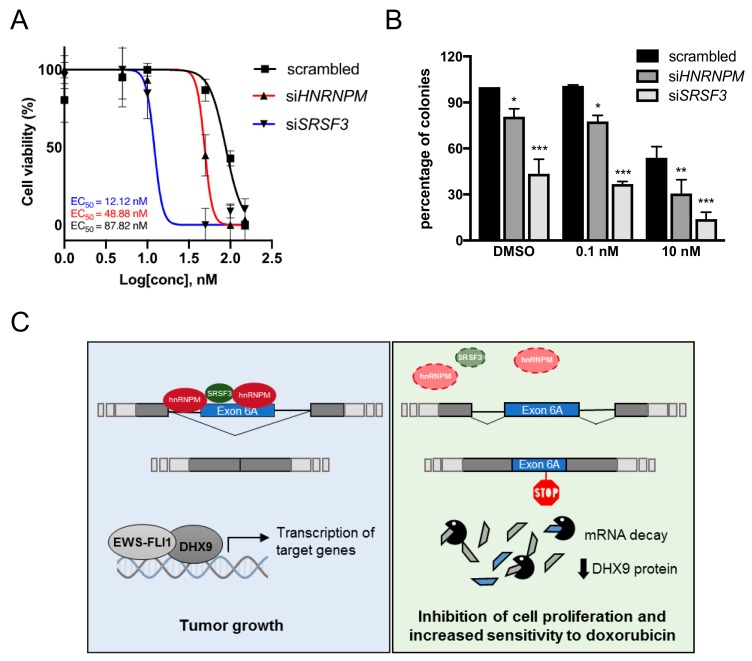
Depletion of *SRSF3* and *hnRNPM* increases doxorubicin sensitivity of Ewing sarcoma cells. **(A**) Dose-response curve of scrambled (black), *siHNRNPM* (red), and *siSRSF3* (blue) TC-71 cells after treatment with increasing concentration of doxorubicin (from 0 to 150 nM). Cells were collected at 72 h after treatment. EC_50_ values are reported on the bottom. (**B**) Colony assay of scrambled, *siSRSF3,* and *siHNRNPM* TC-71 cells treated with increasing concentration of doxorubicin (from 0.1 nM to 10 nM in DMSO). Histogram shows the percentage of colonies determined 12 days after treatment. Statistical analysis was performed by two-way ANOVA. Asterisks indicate significance with Bonferroni post-hoc test (*p*-value: ***< 0.001, ** < 0.01, *< 0.05). (**C**) Graphical representation of the hypothetical regulatory mechanism driving *DHX9* alternative splicing. On the left, hnRNPM and SRSF3 bind *DHX9* pre-mRNA to induce the skipping of exon 6A. The translated full-length DHX9 protein can interact with EWS-FLI1 to promote transcription of target genes involved in cell proliferation and transformation. On the right, in the absence of either SRSF3 or hnRNPM, *DHX9* pre-mRNA is processed to the exon 6a-included noncoding transcript targeted to NMD. The consequent reduction in DHX9 protein levels impacts Ewing sarcoma cells proliferation and sensitizes Ewing sarcoma cells to doxorubicin treatment.

DHX9 helicase has been associated to cell protection from genome instability [11,12,16,17,29,30]. To test whether *SRSF3* and *HNRNPM* depletion had an effect on Ewing sarcoma sensitivity to chemotherapeutic agents, we treated TC-71 cells with doxorubicin. This drug is commonly used in the clinic as a genotoxic agent and is included in the chemotherapeutic regimen for Ewing sarcoma patients [31]. Doxorubicin induces DNA double-strand breaks and can cause a nonreversible checkpoint arrest or trigger cell death, thus curbing the rapid proliferation of cancer cells. MTS assays showed a statistically significant decrease in the viability of TC-71 cells silenced for *HNRNPM* (red) and *SRSF3* (blue) versus control (black) (Figure 6A). In particular, the EC_50_ for doxorubicin activity was 87.82 nM in control transfected TC-71 cells, whereas it was reduced to 48.88 nM and 12.12 nM in si*HNRNPM* and si*SRSF3* cells, respectively (Figure 6A). Similar results were obtained in SK-N-MC (Supplementary Figure S4A), whereas LAP-35 Ewing sarcoma cells displayed only a slight decrease in the EC_50_, in line with the more modest effect on *DHX9* exon 6A inclusion (Supplementary Figure S4B). To validate these results by a different assay, we performed clonogenic assays in TC-71 cells exposed to an increasing amount of doxorubicin (from 0.1 nM to 10 nM). The knockdown of hnRNPM and SRSF3 significantly reduced the ability of TC-71 cells to form colonies in the presence of the drug (Figure 6B). In particular, at 10 nM concentration, we observed a reduction in the percentage of colonies from 54% for control, to 37.5% and 32% for *siHNRMPM* and *siSRSF3*, respectively.

Collectively these results unveil a novel role for SRSF3 and hnRNPM in the regulation of DHX9 alternative splicing, which also impacts on Ewing sarcoma cell sensitivity to chemotherapeutic treatments.

## 4. Discussion

Ewing sarcoma treatment relies on a multidisciplinary approach that combines multi-drug chemotherapy with surgery and local radiation therapy. Such treatments effectively reduce the risk of recurrence and increase the overall survival of patients to 65%. However, pediatric patients have to face severe long-term toxicities due to these invasive therapies. Thus, the development of tailored therapies based on new valuable prognostic markers and therapeutic targets is urgently needed. In this regard, the splicing signature of human cancers is emerging as a powerful tool to distinguish tumor subtypes and stratify patients [32,33]. Notably, although alternative splicing dysregulation has also been reported in Ewing sarcoma [24,34,35,36,37,38], limited information is available regarding the RBPs responsible for this process and their possible association with prognosis. An important regulator of RNA metabolism with strong implications in Ewing sarcoma malignancy is the DNA/RNA helicase DHX9, which interacts with the oncogene EWS-FLI1 and promotes its transcriptional activity. Herein, by querying Ewing sarcoma datasets of patients, we found that the expression of DHX9 is positively associated with disease progression and worse prognosis. Notably, DHX9 interacts with several splicing factors and is itself regulated by alternative splicing [10,11]. Indeed, the inclusion of a poison-exon in the *DHX9* transcript targets it to NMD and lowers DHX9 expression level [11]. Nevertheless, the mechanism that suppresses the inclusion of this poison exon and supports high DHX9 expression in Ewing sarcoma cells was unknown. In this study, by employing multiple approaches, we have now identified several splicing factors (SRSF1, SRSF3, and SRSF10, hnRNPK, hnRNPM, and FUS) as regulators of *DHX9* splicing in Ewing sarcoma cells. Moreover, our work indicates that the silencing of hnRNPM and SRSF3, the strongest repressors of exon 6A, significantly reduces DHX9 expression and Ewing sarcoma cell survival, thus suggesting the functional relevance of their effects on DHX9 splicing regulation.

In our study, we focused on members of the SR proteins and hnRNPs families that were previously shown to play oncogenic functions [27,39,40,41]. Bioinformatic analysis of exon 6A and flanking introns identified the presence of putative binding sites for all the SR and hnRNP proteins that displayed a significant regulation of *DHX9* exon 6A splicing. CLIP experiments confirmed the binding of SRSF1, SRSF3, hnRNPK, hnRNPM, and FUS to the exon 6A of *DHX9* (Figure 3). We also found that the expression of hnRNPM and SRSF3, which showed the strongest repression of exon 6A inclusion, is highly associated with the expression of DHX9 in Ewing sarcoma patients. Remarkably, silencing of *SRSF3* and *HNRNPM*, impacts on DHX9 expression as well as on Ewing sarcoma cell viability and proliferation, while it reduces survival of Ewing sarcoma cells to doxorubicin treatment.

Several studies have previously implicated hnRNPM and SRSF3 in human cancers. hnRNPM is a well-known splicing factor that binds to GU-rich cis-elements in target RNAs [42,43,44,45]. In breast cancer, hnRNPM contributes to metastasis by activating alternative splicing changes that promote epithelial–mesenchymal transition (EMT) [46]. In Ewing sarcoma, we previously documented that the inhibition of the mTOR/AKT/PI3K pathway sets in motion an hnRNPM-dependent alternative splicing program that limits the therapeutic efficacy of these pharmacologic inhibitors [24]. On this basis, we indicated hnRNPM as a new potential therapeutic target to counteract Ewing sarcoma malignancy [24]. Notably, our transcriptomic analysis upon inhibition of the mTOR/AKT/PI3K pathway documented the upregulation of both DHX9 and hnRNPM, and downregulation of *DHX9* exon 6A [24], suggesting a direct regulation of *DHX9* alternative splicing by hnRNPM. Herein, we demonstrate that hnRNPM directly binds to intron 5 and exon 6A in the *DHX9* pre-mRNA, resulting in repression of exon 6A inclusion and stabilization of DHX9 expression. Thus, our results suggest that hnRNPM contributes to Ewing sarcoma malignancy by promoting the high expression of a key regulator of the EWS-FLI1 oncogene.

SRSF3 is the smallest member of the SR family of proteins and plays important roles in the regulation of RNA metabolism, including splicing [47], RNA export [48] and polyadenylation [49], protein translation [50,51], pri-miRNA processing [52], and genome stability [53]. SRSF3 is frequently upregulated in cancer and displays pro-oncogenic functions [54,55,56], whereas tumors displaying reduced SRSF3 expression exhibited slower growth and higher apoptosis [56]. Accordingly, SRSF3 depleted cells were less tumorigenic in nude mice [56]. In particular, SRSF3 was shown to regulate the alternative splicing of genes related to cell proliferation and cell cycle progression in osteosarcoma [55]. Our study now suggests an oncogenic role of SRSF3 also in Ewing sarcoma cells. SRSF3 binds and regulates the *DHX9* exon 6A similarly to hnRNPM, thus also promoting DHX9 expression. Notably, the effect of SRSF3 knockdown on DHX9 protein expression and Ewing sarcoma cell viability was stronger than that elicited by hnRNPM, although displaying a milder effect on the inclusion of the alternative poison exon. This apparent discrepancy could be explained by a direct role played by SRSF3 in the NMD process. Indeed, it was previously shown that SRSF3 depletion in pluripotent cells leads to the downregulation of hundreds of mRNAs that are also regulated at the level of NMD, suggesting a link between SRSF3 and mRNA surveillance pathway [57,58]. However, the exact role of SRSF3 in NMD has not been elucidated yet.

Our study documents that SRSF3 and hnRNPM downregulation reduces Ewing sarcoma cell proliferation and increases Ewing sarcoma sensitivity to doxorubicin treatment (Figure 6). Ewing sarcoma patients are generally treated with a potent cocktail of five drugs, including doxorubicin. Thus, targeting SRSF3 and hnRNPM expression, could have a potential therapeutic value by sensitizing cells to drug treatment, and could, therefore, be exploited in a combination therapy. *SRSF3* depletion displayed a stronger effect than *HNRNPM* depletion on Ewing sarcoma sensitivity to chemotherapy treatment, in line with its stronger impact on DHX9 expression. Notably, the chromosome region bearing the *SRSF3 locus* on chromosome 6p is commonly amplified in cancer [59], thus causing aberrant SRSF3 overexpression. Moreover, the downregulation of SRSF3 by antisense oligonucleotides (ASO) was shown to sensitize oral squamous cell carcinoma and breast cancer cells to paclitaxel treatment [60]. These findings suggest that the depletion of SRSF3 expression may represent a valuable therapeutic tool also for Ewing sarcoma. Noteworthy, although we focused on the DHX9 poison-exon as a splicing target of SRSF3 and hnRNPM with direct relevance for Ewing sarcoma, our findings do not rule out possible effects of these RBPs on other splicing variants of the same or other genes in this disease.

## 5. Conclusions

In conclusion, our study shows that DHX9 expression in Ewing sarcoma correlates with worse outcome in patients. SRSF3 and hnRNPM regulate poison-exon inclusion in DHX9 and sensitize Ewing sarcoma cells to chemotherapy. Thus, we suggest that modulation of SRSF3 and hnRNPM expression and their splicing signature could represent a novel therapeutic opportunity for combined and less aggressive therapy in Ewing sarcoma.

## Supplementary Materials

The following are available online at https://www.mdpi.com/2073-4409/9/2/328/s1, Supplementary Figure S1 RT-qPCR analysis to monitor the inclusion of DHX9 exon 6A normalized to the skipped isoform (junction exon 6-exon 7), upon knockdown of the indicated SR (A) and hnRNP (B) proteins, Supplementary Figure S2 Pearson correlation analysis on Ewing sarcoma patients, between the expression of DHX9 and either hnRNPL (A) or SRSF5 (B), Supplementary Figure S3. HNRNPM and SRSF3 affect DHX9 exon 6A inclusion in SK-N-MC and LAP-35 Ewing sarcoma cells, Supplementary Figure S4. Depletion of SRSF3 and hnRNPM increases doxorubicin sensitivity of SK-N-MC and LAP-35 Ewing sarcoma cells, Supplementary Table S1 List of oligonucleotides used in the siRNA library, Supplementary Table S2 List of primers.
