# Supplementary material for: Poison-Exon Inclusion in DHX9 Reduces Its Expression and Sensitizes Ewing Sarcoma Cells to Chemotherapeutic Treatment
Source: Cells. 2020 Jan 31;9(2):328. doi: 10.3390/cells9020328 (PMC7072589; doi:10.3390/cells9020328)
Supplement: Supplementary file 1 [file cells-09-00328-s001.pdf]

Appendix A

Poison-exon inclusion in DHX9 reduces its expression and sensitizes Ewing sarcoma cells to chemotherapeutic treatment

Ramona Palombo[1], Veronica Verdile[1,2] and Maria Paola Paronetto[1,2,*]

[1] Laboratory of Cellular and Molecular Neurobiology, IRCCS Fondazione Santa Lucia, Rome, Italy
[2] Università degli Studi di Roma "Foro Italico", Piazza Lauro de Bosis, 15, Rome, Italy
* Correspondence: mariapaola.paronetto@uniroma4.it;

Supplementary Figure Legends 1-4

Supplementary Figures 1-4

Supplementary Table 1. List of oligonucleotides used in the siRNA library.

Supplementary Table 2. List of primers.

Supplementary Figure 1. RT-qPCR analysis to monitor the inclusion of *DHX9* exon 6A normalized to the skipped isoform (junction exon 6-exon 7), upon knockdown of the indicated SR (**A**) and hnRNP (**B)** proteins. The reported values are the average (± S.D.) of three independent experiments and statistical analysis was performed by Student *t*-test comparing each siRNA to scrambled. (*p*-value: ***<0.001, ** <0.01, *<0.05). (C) Western blot analysis to monitor the protein levels of SRSF1, SRSF3, hnRNPM, hnRNPK and hnRNPP (FUS) upon treatment with specific siRNA oligonucleotides. 20μg of total extracts were loaded on a 10% acrylamide gels. Histograms show the relative levels of the RBPs normalized to β-actin. The reported values are the average (± S.D.) of three independent experiments and statistical analysis was performed by Student *t*-test comparing each siRNA to scrambled (*p*-value: ***<0.001, ** <0.01, *<0.05). (D) Western blot analysis to monitor the protein levels of EWS and EWS-FLI1 upon FUS knockdown, normalized to β-actin. The histogram on the right shows the densitometric analyses of three independent experiments. Statistical analysis was performed by Student *t*-test (*p*-value: *<0.05).

Supplementary Figure 2. Pearson correlation analysis on Ewing sarcoma patients, between the expression of DHX9 and either hnRNPL (A) or SRSF5 (B). Values are expressed as logarithmic with base 2. In each panel is reported the correlation value (R) and the relative *p*-value.

Supplementary Figure 3. HNRNPM and SRSF3 affects *DHX9* exon 6A inclusion in Ewing sarcoma. (A) and (B) RT-qPCR to monitor *DHX9* exon 6A inclusion in SK-N-MC (A) and LAP-35 (B) Ewing sarcoma cells, as indicated, upon knockdown of *HNRNPM* and *SRSF3*. (C) and (D) Western blot analysis upon transfection of scrambled, siHNRNPM or siSRSF3 oligonucleotides in SK-N-MC and LAP-35 Ewing sarcoma cells. The reported values are the average (± S.D.) of three independent experiments and statistical analysis was performed by Student *t*-test comparing each siRNA to scrambled (*p*-value: ***<0.001, ** <0.01, *<0.05).

Supplementary Figure 4. Depletion of SRSF3 and hnRNPM increases doxorubicin sensitivity of Ewing sarcoma cells. (A) and (B) Dose-response curves of scrambled (black), *siHNRNPM* (red) and *siSRSF3* (blue) in SK-N-MC (A) and LAP-35 (B) Ewing sarcoma cells after treatment with increasing concentration of doxorubicin (from 0 to 150 nM). Cells were collected at 72 hours after treatment. EC_{50} values are reported on the bottom.

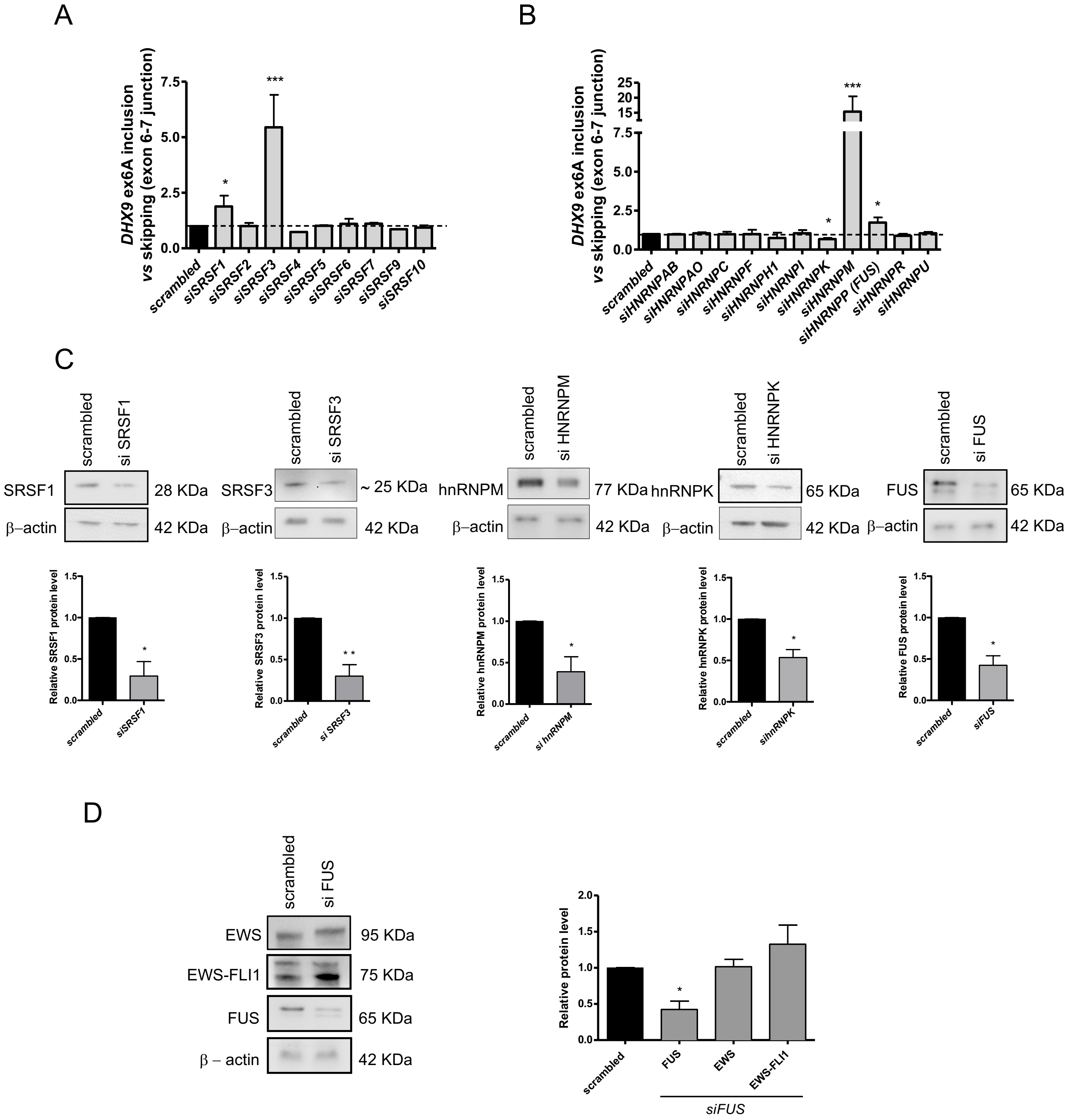
A
DHX9 ex6A inclusion vs skipping (exon 6-7 junction)
7.5
5.0
2.5
0.0
*
***
scrambled
siSRSF1
siSRSF2
siSRSF3
siSRSF4
siSRSF5
siSRSF6
siSRSF7
siSRSF9
siSRSF10
B
DHX9 ex6A inclusion vs skipping (exon 6-7 junction)
25
20
15
7.5
5.0
2.5
0.0
*
***
*
scrambled
siHNRNPAB
siHNRNPAO
siHNRNPC
siHNRNPF
siHNRNPH1
siHNRNPI
siHNRNPK
siHNRNPM
siHNRNPP (FUS)
siHNRNPR
siHNRNPU
C
scrambled
si SRSF1
SRSF1
28 KDa
β–actin
42 KDa
scrambled
si SRSF3
SRSF3
~ 25 KDa
β–actin
42 KDa
scrambled
si HNRNPM
hnRNPM
77 KDa
β–actin
42 KDa
scrambled
si HNRNPK
hnRNPK
65 KDa
β–actin
42 KDa
scrambled
si FUS
FUS
65 KDa
β–actin
42 KDa
Relative SRSF1 protein level
scrambled
siSRSF1
Relative SRSF3 protein level
scrambled
si SRSF3
Relative hnRNPM protein level
scrambled
si hnRNPM
Relative hnRNPK protein level
scrambled
sihnRNPK
Relative FUS protein level
scrambled
siFUS
D
scrambled
si FUS
EWS
95 KDa
EWS-FLI1
75 KDa
FUS
65 KDa
β – actin
42 KDa
Relative protein level
2.0
1.5
1.0
0.5
0.0
*
scrambled
FUS
EWS
EWS-FLI1
siFUS

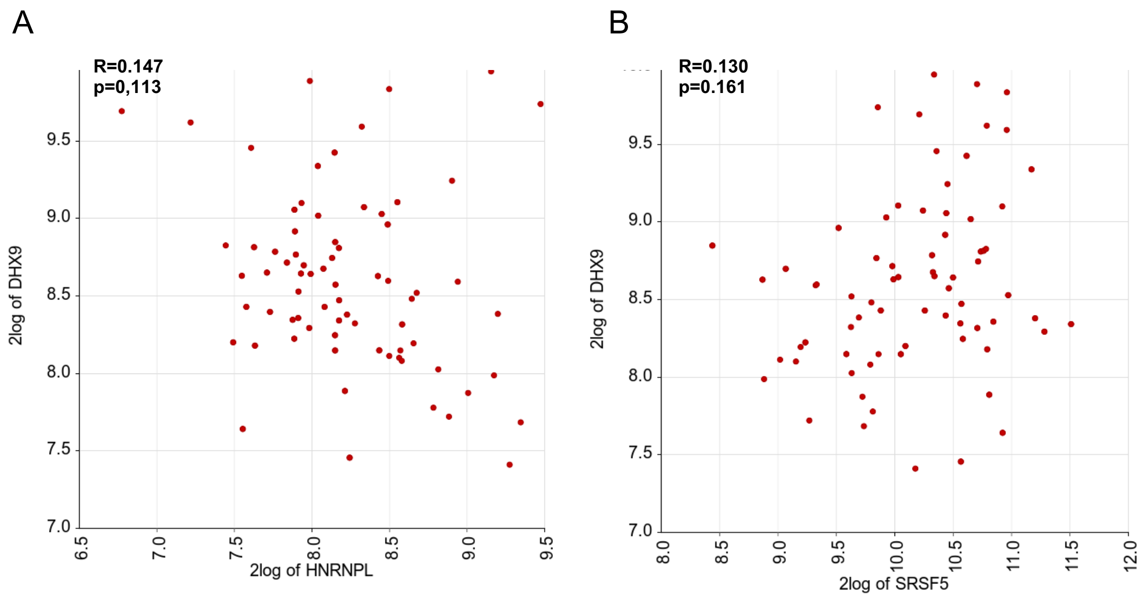
A
R=0.147
p=0,113
2log of DHX9
2log of HNRNPL
B
R=0.130
p=0.161
2log of DHX9
2log of SRSF5

Supplementary Figure 3

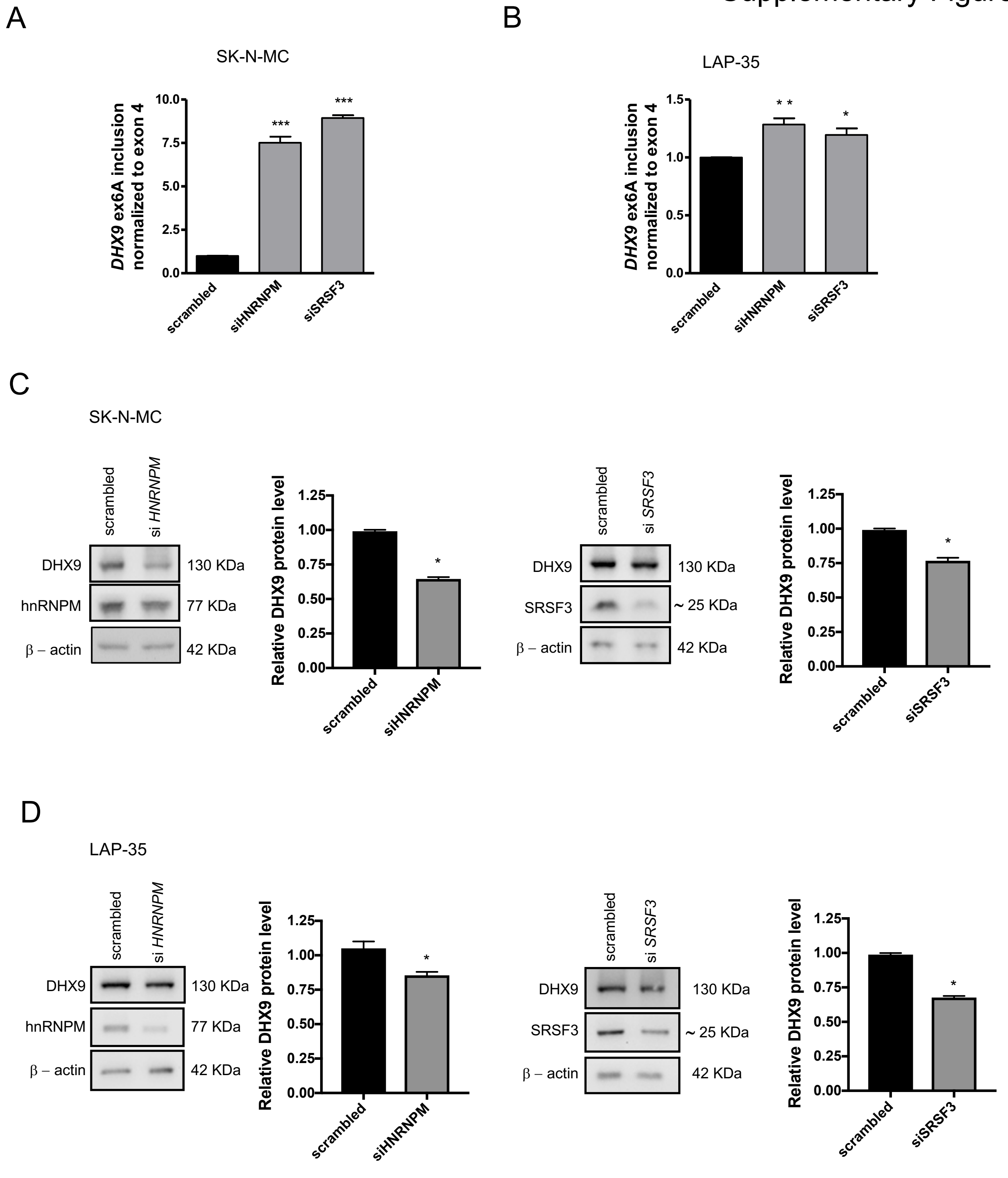

Supplementary Figure 4

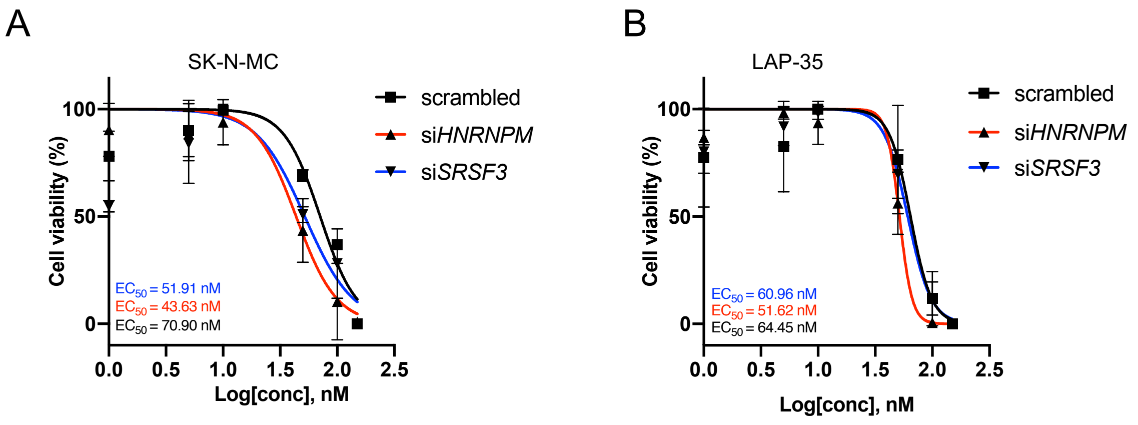

Supplementary Table 1: siRNAs

Mission siRNA (SIGMA)	Cod.
siHNRNPAB	SASI_Hs01_00048471
siHNRNPAO	SASI_Hs01_00149990
siHNRNPC	SASI_Hs02_00312564
siHNRNPF	SASI_Hs02_00318648
siHNRNPH1	SASI_Hs01_00113607
siHNRNPI	SASI_Hs01_00216643
siHNRNPR	SASI_Hs02_00323053
siHNRNPU	SASI_Hs01_00063626
siSRSF1	SASI_Hs02_00313260
siSRSF2	SASI_Hs01_00081729
siSRSF3	SASI_Hs01_00018995
siSRSF4	SASI_Hs01_00065340
siSRSF5	SASI_Hs01_00103981
siSRSF6	SASI_Hs02_00341239
siSRSF7	SASI_Hs01_00094911
siSRSF9	SASI_Hs01_00220732
siSRSF10	SASI_Hs01_00162140
Universal negative control #1	SIC001
**siRNA (Santa Cruz Biotechnology)**	**Cod.**
siHNRNPK	sc-38282
siHNRNPM	sc-38286
siHNRNPP	sc-40563

Supplementary Table 2. List of primers.

Primer	Sequence 5’- 3’
β-actin_FW	CATGGAGTCCTGTGGCATC
β-actin_REV	AGCACTGTGTTGGCGTACAG
CCND1_FW	GTGCAAGGCCTGAACCTG
CCND1_REV	CGGGTCACACTTGATCACTC
c-MYC_FW	CTCCACCTCCAGCTTGTACC
c-MYC_REV	GCTGTCGTTGAGAGGGTAGG
DHX9_EX4 FW gene expression	ATCTCCGCCCCCACTTACT
DHX9_EX4 REV gene expression	GAGAGCCAGATGTGGAGGAA
DHX9_EX6A FW alternative splicing	GTCCTGATCACAACAGGTGG
DHX9_EX6A REV alternative splicing	GCTTTCAGGGGAACAACATC
DHX9_EX6A FW CLIP	GGAGGAGAATGAGATTGAGTGC
DHX9_EX6A REV CLIP	GCTTTCAGGGGAACAACATC
DHX9_i6 FW CLIP	TTATTACTCCCAGGAAGGC
DHX9_i6 FW CLIP	GGAATCATTCAAAAAGGTTTCC
EZH2_FW	ATAACAGTAGCAGGCCCAGC
EZH2_REV	GTTTCAGTCCCTGCTTCCCT
hnRNPAB_FW	GCTACGACTACTCGCCCTATG
hnRNPAB_REV	CGATCAGTTGGTCGCTCCTG
hnRNPAO_FW	AGGCCGAGATTATTGCCGAC
hnRNPAO_REV	TATCCTCCTTGGGGACTGCT
hnRNPC_FW	CACCCTTCCTGGGTGATGTT
hnRNPC_REV	TGAACAGAGCAGCCCACAAT
hnRNPF_FW	CCGCCAAAACACGTTTACCG

hnRNPF_REV	GGGAAACTCCAGGTGCATGT
hnRNPH1_FW	CAGTTCAGCGACCACGTTTG
hnRNPH1_REV	CACCACGAATCCCTCTCCAC
hnRNPI_FW	TTTTCCAAGCTCACCAGCCT
hnRNPI_REV	TATACCAGGTGCACCGAAGG
hnRNPK_FW	CGCTCGTTTTCTGTCTAGCTC
hnRNPK_REV	ACAACTGACACCCCAGTGC
hnRNPM_FW	AGTATGGCTGGTGTGGTGGT
hnRNPM_REV	TTGCACAGCTTCAATGGACT
hnRNPP_FW	AGGCCTGGGTGAGAATGTTA
hnRNPP_REV	GCTGTCCCGTTTTCTTGTTT
hnRNPR_FW	TCAGGTGAATGGTAATGCGGT
hnRNPR_REV	GGCCTGCCTCTATCAGTGTC
hnRNPU_FW	CAGCAGCAGGCGGGAG
hnRNPU_REV	GTGGCTGAGGAGATTTGGCT
ID2_FW	CCCAGAACAAGAAGGTGAGC
ID2_REV	ATAGTGGGATGCGAGTCCAG
NR0B1_FW	CCAGGTCCAAGCCATCAAGT
NR0B1_REV	TAAAGAGCACGGTCCCCTTG
SRSF1_FW	CCGCATCTACGTGGGTAACT
SRSF1_REV	GCGATTCTTGAGGTCGATGT
SRSF2_FW	TCCAAGTCCAAGTCCTCGTC
SRSF2_REV	TTGGATTCCCTCTTGGACAC
SRSF3_FW	TTGGAACGGGCTTTTGGCTA
SRSF3_REV	CCATCTAGCTCTCGGACTGC

SRSF4_FW	CAAGCCAGGTTCCAGACGAC
SRSF4_REV	CGGGAATGTCTGCTTCGAGA
SRSF5_FW	GAAGGGGTGGTTGAGTTTGC
SRSF5_REV	CGGGATCGAGACCTGCTTC
SRSF6_FW	GGTCACGAAGTAGGAGTCGC
SRSF6_REV	ACGTGATCGACCTTTGCTCC
SRSF7_FW	GCAGAAGATGCAGTACGAGG
SRSF7_REV	TCGGGCAGGTGGTCTATCAA
SRSF9_FW	TATGCCCTGCGTAAACTGGA
SRSF9_REV	CCAGACCGAGACCGTGAGTA
SRSF10_FW	AGCCAAGGAAGGGAGGAATGT
SRSF10_REV	ACCGACTTCTTGATCTCCTCCT
